# *N*,*N*-Dimethyl-*N*-propyl­propan-1-aminium chloride monohydrate

**DOI:** 10.1107/S1600536808032340

**Published:** 2008-10-11

**Authors:** Minna Kärnä, Manu Lahtinen, Jussi Valkonen

**Affiliations:** a University of Jyväskylä, Department of Chemistry, PO Box 35, FIN-40014 JY, Finland

## Abstract

The title compound, C_8_H_20_N^+^·Cl^−^·H_2_O, has been prepared by a simple one-pot synthesis route followed by anion exchange using resin. In the crystal structure, the cations are packed in such a way that channels exist parallel to the *b* axis. These channels are filled by the anions and water mol­ecules, which inter­act *via* O—H⋯Cl hydrogen bonds [O⋯Cl = 3.285 (3) and 3.239 (3) Å] to form helical chains. The cations are involved in weak inter­molecular C—H⋯Cl and C—H⋯O hydrogen bonds. The title compound is not isomorphous with the bromo or iodo analogues.

## Related literature

For general background, see: Ropponen *et al.* (2004 ▶). For related structures, see: Busi *et al.* (2005 ▶).
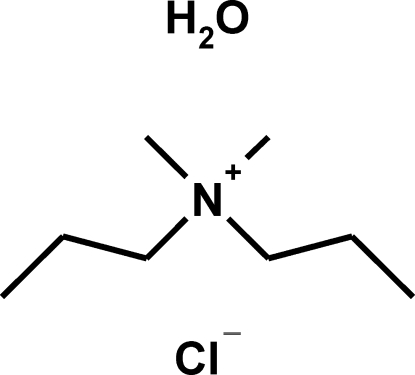


## Experimental

### 

#### Crystal data

C_8_H_20_N^+^·Cl^−^·H_2_O
*M* *_r_* = 183.72
Monoclinic, 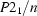

*a* = 7.9870 (16) Å
*b* = 9.4210 (19) Å
*c* = 14.875 (3) Å
β = 100.23 (3)°
*V* = 1101.5 (4) Å^3^
*Z* = 4
Cu *K*α radiation
μ = 2.71 mm^−1^
*T* = 173 (2) K
0.40 × 0.12 × 0.12 mm

#### Data collection

Nonius Kappa APEXII diffractometer
Absorption correction: multi-scan (*SADABS*; Sheldrick, 2004 ▶) *T*_min_ = 0.534, *T*_max_ = 0.737
6471 measured reflections
1784 independent reflections
1474 reflections with *I* > 2σ(*I*)
*R*_int_ = 0.056

#### Refinement

*R*[*F* ^2^ > 2σ(*F* ^2^)] = 0.042
*wR*(*F* ^2^) = 0.108
*S* = 1.04
1784 reflections
112 parameters
H atoms treated by a mixture of independent and constrained refinement
Δρ_max_ = 0.20 e Å^−3^
Δρ_min_ = −0.21 e Å^−3^

### 

Data collection: *COLLECT* (Nonius, 2002 ▶); cell refinement: *DENZO–SMN* (Otwinowski & Minor, 1997 ▶; Otwinowski *et al.*, 2003 ▶); data reduction: *DENZO–SMN*; program(s) used to solve structure: *SIR2002* (Burla *et al.*, 2003 ▶); program(s) used to refine structure: *SHELXL97* (Sheldrick, 2008 ▶); molecular graphics: *DIAMOND* (Brandenburg, 2008 ▶); software used to prepare material for publication: *SHELXL97*.

## Supplementary Material

Crystal structure: contains datablocks I, global. DOI: 10.1107/S1600536808032340/cv2460sup1.cif

Structure factors: contains datablocks I. DOI: 10.1107/S1600536808032340/cv2460Isup2.hkl

Additional supplementary materials:  crystallographic information; 3D view; checkCIF report

## Figures and Tables

**Table 1:** Hydrogen-bond geometry (Å, °)

*D*—H⋯*A*	*D*—H	H⋯*A*	*D*⋯*A*	*D*—H⋯*A*
O1*W*—H1*W*⋯Cl1	0.79 (4)	2.47 (4)	3.239 (3)	164 (3)
O1*W*^i^—H2*W*^i^⋯Cl1	0.84 (4)	2.46 (4)	3.285 (3)	172 (4)
C31^ii^—H5*B*^ii^⋯O1*W*	0.98	2.54	3.489 (4)	162
C21^ii^—H4*A*^ii^⋯Cl1	0.99	2.76	3.742 (2)	172
C41^iii^—H7*A*^iii^⋯Cl1	0.98	2.80	3.721 (3)	156
C41—H7*C*⋯Cl1	0.98	2.76	3.691 (3)	158

Symmetry codes: (i) 
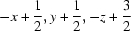
; (ii) 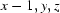; (iii) 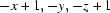.

**Fig. 1.**
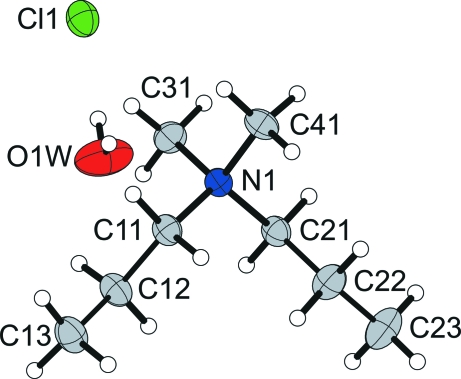
The molecular structure of (1) showing the atomic numbering and 50% probability displacement ellipsoids.

**Fig. 2.**
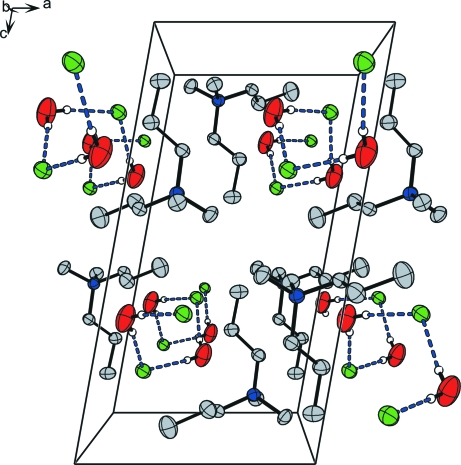
The packing of (1) viewed along the crystallographic b-axis. Dashed lines indicate hydrogen bonds. The helical structure of the network between the anions and the water molecules can be seen. The H atoms not involved in the network have been omitted for clarity, as well as some of the cations.

## supplementary crystallographic information

### Comment

As a part of our ongoing study of small *R*_2_R'_2_N^+^*X*^-^-type quaternary ammonium halides (Ropponen *et al.*, 2004; Busi *et al.*, 2005) the title compound (Fig. 1) has been synthesized and its crystal structure is reported here.

The asymmetric unit consists of one cation and one anion with one water molecule. The intermolecular (O)H···Cl distances vary from 2.456 (41) to 2.477 (40) Å. The shortest intermolecular (C)H···Cl distance is 2.779 (1) Å and the shortest (C)H···O distance is 2.561 (3) Å. The packing is affected by these weak intermolecular bonds (Table 1) causing the cations to arrange in layers which are separated by anions and the water molecules. The anions and the water molecules form a hydrogen-bonded chain along the crystallographic *b-*axis.

### Experimental

The mixture of 1-bromopropane (95.2 mmol) and dimethylformamide (0.47 mol) in the presence of potassiumcarbonate (95.2 mmol) was stirred at 70°C for 72 h. The reaction mixture was cooled and filtered and the filtrate was evaporated. The product (white powder) was washed with diethyl ether and recrystallized from dichloromethane and dried *in vacuo*. The anion exhange was performed in a suitable resin, resulting in a light yellow, hygroscopic final product.

### Refinement

The water H atoms were located from the difference Fourier map and refined isotropically. Other H atoms were positioned geometrically and refined using a riding model with C—H = 0.98–0.99 Å and with *U*ĩso~(H) = 1.2 (1.5 for methyl groups) times *U*~eq~(C).

### Crystal data

**Table:**

C_8_H_20_N^+^·Cl^−^·H_2_O	*F*(000) = 408
*M**_r_* = 183.72	*D*_x_ = 1.108 Mg m^−^^3^
Monoclinic, *P*2_1_/*n*	Cu *K*α radiation, λ = 1.54178 Å
*a* = 7.9870 (16) Å	Cell parameters from 1705 reflections
*b* = 9.4210 (19) Å	θ = 0.9–63.7°
*c* = 14.875 (3) Å	µ = 2.71 mm^−^^1^
β = 100.23 (3)°	*T* = 173 K
*V* = 1101.5 (4) Å^3^	Rod, colourless
*Z* = 4	0.40 × 0.12 × 0.12 mm

### Data collection

**Table:**

Nonius Kappa APEXII diffractometer	1784 independent reflections
Radiation source: fine-focus sealed tube	1474 reflections with *I* > 2σ(*I*)
graphite	*R*_int_ = 0.056
φ and ω scans	θ_max_ = 63.4°, θ_min_ = 5.6°
Absorption correction: multi-scan (SADABS; Sheldrick, 2004)	*h* = −7→9
*T*_min_ = 0.534, *T*_max_ = 0.737	*k* = −10→10
6471 measured reflections	*l* = −17→16

### Refinement

**Table:**

Refinement on *F*^2^	Primary atom site location: structure-invariant direct methods
Least-squares matrix: full	Secondary atom site location: difference Fourier map
*R*[*F*^2^ > 2σ(*F*^2^)] = 0.042	Hydrogen site location: inferred from neighbouring sites
*wR*(*F*^2^) = 0.108	H atoms treated by a mixture of independent and constrained refinement
*S* = 1.04	*w* = 1/[σ^2^(*F*_o_^2^) + (0.0535*P*)^2^ + 0.2799*P*] where *P* = (*F*_o_^2^ + 2*F*_c_^2^)/3
1784 reflections	(Δ/σ)_max_ < 0.001
112 parameters	Δρ_max_ = 0.20 e Å^−^^3^
0 restraints	Δρ_min_ = −0.21 e Å^−^^3^

### Special details

**Table:**

Geometry. All e.s.d.'s (except the e.s.d. in the dihedral angle between two l.s. planes) are estimated using the full covariance matrix. The cell e.s.d.'s are taken into account individually in the estimation of e.s.d.'s in distances, angles and torsion angles; correlations between e.s.d.'s in cell parameters are only used when they are defined by crystal symmetry. An approximate (isotropic) treatment of cell e.s.d.'s is used for estimating e.s.d.'s involving l.s. planes.
Refinement. Refinement of *F*^2^ against ALL reflections. The weighted *R*-factor *wR* and goodness of fit *S* are based on *F*^2^, conventional *R*-factors *R* are based on *F*, with *F* set to zero for negative *F*^2^. The threshold expression of *F*^2^ > σ(*F*^2^) is used only for calculating *R*-factors(gt) *etc*. and is not relevant to the choice of reflections for refinement. *R*-factors based on *F*^2^ are statistically about twice as large as those based on *F*, and *R*- factors based on ALL data will be even larger.

### Fractional atomic coordinates and isotropic or equivalent isotropic displacement parameters (Å^2^)

**Table:**

	*x*	*y*	*z*	*U*_iso_*/*U*_eq_	
Cl1	0.33660 (7)	−0.05079 (6)	0.66252 (4)	0.0363 (2)	
N1	0.7874 (2)	0.10789 (19)	0.61585 (11)	0.0244 (4)	
O1W	0.0649 (3)	−0.2974 (3)	0.68554 (19)	0.0673 (7)	
C21	0.9430 (2)	0.1617 (2)	0.58112 (14)	0.0272 (5)	
H4A	1.0416	0.1023	0.6077	0.033*	
H4B	0.9243	0.1489	0.5140	0.033*	
C31	0.7647 (3)	−0.0457 (2)	0.59034 (16)	0.0311 (5)	
H5A	0.6659	−0.0836	0.6131	0.047*	
H5B	0.8668	−0.0987	0.6175	0.047*	
H5C	0.7466	−0.0551	0.5237	0.047*	
C13	0.9427 (3)	0.0851 (3)	0.87601 (15)	0.0366 (6)	
H6A	0.9657	0.1866	0.8863	0.055*	
H6B	1.0303	0.0295	0.9153	0.055*	
H6C	0.8308	0.0622	0.8905	0.055*	
C41	0.6317 (3)	0.1857 (3)	0.56898 (16)	0.0333 (5)	
H7A	0.6236	0.1783	0.5026	0.050*	
H7B	0.6399	0.2859	0.5870	0.050*	
H7C	0.5302	0.1437	0.5868	0.050*	
C11	0.7990 (2)	0.1267 (2)	0.71770 (13)	0.0269 (5)	
H8A	0.6911	0.0936	0.7346	0.032*	
H8B	0.8097	0.2294	0.7319	0.032*	
C12	0.9448 (3)	0.0497 (3)	0.77691 (15)	0.0319 (5)	
H9A	0.9323	−0.0540	0.7672	0.038*	
H9B	1.0542	0.0796	0.7604	0.038*	
C22	0.9876 (3)	0.3154 (2)	0.60251 (17)	0.0360 (6)	
H10A	1.0077	0.3301	0.6695	0.043*	
H10B	0.8915	0.3768	0.5749	0.043*	
C23	1.1457 (3)	0.3563 (3)	0.5653 (2)	0.0497 (7)	
H11A	1.2408	0.2956	0.5929	0.074*	
H11B	1.1739	0.4558	0.5802	0.074*	
H11C	1.1247	0.3438	0.4989	0.074*	
H1W	0.141 (4)	−0.250 (4)	0.675 (2)	0.065 (11)*	
H2W	0.100 (4)	−0.360 (4)	0.724 (3)	0.084 (12)*	

### Atomic displacement parameters (Å^2^)

**Table:**

	*U*^11^	*U*^22^	*U*^33^	*U*^12^	*U*^13^	*U*^23^
Cl1	0.0368 (3)	0.0399 (4)	0.0335 (3)	0.0034 (2)	0.0099 (2)	0.0041 (2)
N1	0.0251 (9)	0.0244 (10)	0.0234 (9)	0.0008 (7)	0.0037 (7)	0.0005 (7)
O1W	0.0431 (12)	0.0528 (15)	0.1006 (19)	−0.0032 (11)	−0.0020 (12)	0.0265 (13)
C21	0.0270 (11)	0.0327 (13)	0.0226 (11)	0.0014 (9)	0.0067 (8)	0.0011 (9)
C31	0.0359 (12)	0.0241 (12)	0.0326 (12)	−0.0012 (9)	0.0038 (9)	−0.0041 (9)
C13	0.0479 (14)	0.0344 (14)	0.0265 (12)	0.0043 (11)	0.0038 (10)	−0.0001 (10)
C41	0.0264 (11)	0.0375 (14)	0.0333 (13)	0.0071 (10)	−0.0020 (9)	0.0037 (10)
C11	0.0281 (11)	0.0305 (13)	0.0232 (11)	0.0028 (9)	0.0074 (8)	0.0002 (9)
C12	0.0321 (11)	0.0384 (14)	0.0251 (12)	0.0046 (10)	0.0047 (9)	0.0023 (10)
C22	0.0391 (12)	0.0319 (14)	0.0390 (14)	−0.0060 (10)	0.0122 (10)	−0.0033 (10)
C23	0.0465 (14)	0.0420 (16)	0.0639 (18)	−0.0122 (12)	0.0192 (13)	0.0043 (13)

### Geometric parameters (Å, °)

**Table:**

N1—C31	1.499 (3)	C13—H6C	0.9800
N1—C41	1.504 (3)	C41—H7A	0.9800
N1—C11	1.512 (3)	C41—H7B	0.9800
N1—C21	1.515 (3)	C41—H7C	0.9800
O1W—H1W	0.79 (4)	C11—C12	1.514 (3)
O1W—H2W	0.84 (4)	C11—H8A	0.9900
C21—C22	1.512 (3)	C11—H8B	0.9900
C21—H4A	0.9900	C12—H9A	0.9900
C21—H4B	0.9900	C12—H9B	0.9900
C31—H5A	0.9800	C22—C23	1.516 (3)
C31—H5B	0.9800	C22—H10A	0.9900
C31—H5C	0.9800	C22—H10B	0.9900
C13—C12	1.514 (3)	C23—H11A	0.9800
C13—H6A	0.9800	C23—H11B	0.9800
C13—H6B	0.9800	C23—H11C	0.9800
			
C31—N1—C41	107.48 (16)	N1—C41—H7C	109.5
C31—N1—C11	110.50 (16)	H7A—C41—H7C	109.5
C41—N1—C11	107.79 (16)	H7B—C41—H7C	109.5
C31—N1—C21	107.86 (15)	N1—C11—C12	115.50 (16)
C41—N1—C21	109.83 (16)	N1—C11—H8A	108.4
C11—N1—C21	113.22 (15)	C12—C11—H8A	108.4
H1W—O1W—H2W	110 (3)	N1—C11—H8B	108.4
C22—C21—N1	115.22 (17)	C12—C11—H8B	108.4
C22—C21—H4A	108.5	H8A—C11—H8B	107.5
N1—C21—H4A	108.5	C13—C12—C11	108.70 (18)
C22—C21—H4B	108.5	C13—C12—H9A	109.9
N1—C21—H4B	108.5	C11—C12—H9A	109.9
H4A—C21—H4B	107.5	C13—C12—H9B	109.9
N1—C31—H5A	109.5	C11—C12—H9B	109.9
N1—C31—H5B	109.5	H9A—C12—H9B	108.3
H5A—C31—H5B	109.5	C21—C22—C23	110.3 (2)
N1—C31—H5C	109.5	C21—C22—H10A	109.6
H5A—C31—H5C	109.5	C23—C22—H10A	109.6
H5B—C31—H5C	109.5	C21—C22—H10B	109.6
C12—C13—H6A	109.5	C23—C22—H10B	109.6
C12—C13—H6B	109.5	H10A—C22—H10B	108.1
H6A—C13—H6B	109.5	C22—C23—H11A	109.5
C12—C13—H6C	109.5	C22—C23—H11B	109.5
H6A—C13—H6C	109.5	H11A—C23—H11B	109.5
H6B—C13—H6C	109.5	C22—C23—H11C	109.5
N1—C41—H7A	109.5	H11A—C23—H11C	109.5
N1—C41—H7B	109.5	H11B—C23—H11C	109.5
H7A—C41—H7B	109.5		
			
C31—N1—C21—C22	−178.85 (18)	C41—N1—C11—C12	177.38 (18)
C41—N1—C21—C22	64.3 (2)	C21—N1—C11—C12	−60.9 (2)
C11—N1—C21—C22	−56.3 (2)	N1—C11—C12—C13	177.40 (18)
C31—N1—C11—C12	60.2 (2)	N1—C21—C22—C23	179.42 (19)

### Hydrogen-bond geometry (Å, °)

**Table:**

*D*—H···*A*	*D*—H	H···*A*	*D*···*A*	*D*—H···*A*
O1W—H1W···Cl1	0.79 (4)	2.47 (4)	3.239 (3)	164 (3)
O1W^i^—H2W^i^···Cl1	0.84 (4)	2.46 (4)	3.285 (3)	172 (4)
C31^ii^—H5B^ii^···O1W	0.98	2.54	3.489 (4)	162
C21^ii^—H4A^ii^···Cl1	0.99	2.76	3.742 (2)	172
C41^iii^—H7A^iii^···Cl1	0.98	2.80	3.721 (3)	156
C41—H7C···Cl1	0.98	2.76	3.691 (3)	158

Symmetry codes:  (i) −*x*+1/2, *y*+1/2, −*z*+3/2; (ii) *x*−1, *y*, *z*; (iii) −*x*+1, −*y*, −*z*+1.
